# The Electrolytic Destruction of Bacilli

**Published:** 1907-10-19

**Authors:** 


### The Electrolytic Destruction of Bacilli.

News comes from Dewsbury that a technical chemist, Mr. Henry Hay, has more or less accidentally discovered a comparatively easy way of rendering wool or rags impregnated with anthrax, and other micro-organisms, entirely germ-free by a process of electrolysis which not only does not damage the wool, but which was actually invented for the purpose of cleansing it from cotton and other similar impurities. Mr. Hay was experimenting with a view to discover how to get rid of the cotton and other vegetable fibres from amongst the tons and tons of rags of all sorts which are used in the manufacture of cheap tweeds and other cloths. We do not propose to criticise this trade; the interest of Mr. Hay's work from our point of view is that, by a simple electrolytic process, he is said to have succeeded in destroying the cotton fibres, leaving the wool intact, and to have incidentally discovered that the wool was at the same time rendered germ-free. This, if true, is a lucky hit, killing two birds with one stone. It is a matter upon which we hope to have more details in due course. Its importance from a public health point of view is great. Wools imported from certain countries are scheduled by the Home Office as requiring special treatment before being manufactured into goods; to get this treatment, the wool has to be conveyed to the places where there are special arrangements for it; there is liability to anthrax infection of persons *en route*, and the present treatment itself damages the wool to some extent and prevents its subsequent use for certain purposes. Mr. Hay's method appears to be so simple that the necessary appliances could be arranged for at the port of importation at small cost; it seems that it is not even necessary to undo the bales of wool; they can be made sterile by casting them into a tank of solution, through which an electric current is passed, the wool at the same time being improved rather than damaged. It is clear that further experiments are needed to see whether what we are told is true or not; and we look forward to hearing that Mr. Hay's discovery receives confirmation from others. The Home Office cannot but be interested in the matter.

The disinfection of woollen things of all sorts after infectious fevers and the like is always a matter of difficulty. Blankets, for example, and suits of clothing are very often greatly damaged when sterilised by moist heat. If the electrolytic process destroys anthrax bacilli and leaves wool fibre absolutely intact, a new light is thrown upon the question how to sterilise woollen things after scarlet fever, for example. If anthrax germs and vegetable fibres are destroyed by a simple process of electrolysis, it seems reasonable to suppose that the infective agents in the specific fevers would be so too. The subject is at least worthy of discussion; if Mr. Hay's contentions are confirmed, experiments might be carried out in this direction. It is not likely that the electrolytic method could be applied in private houses, but in fever hospitals it might be of great service. It would be most necessary, however, to be sure that the woollen goods were all wool and not partly cotton, else the destruction of the latter would inevitably disintegrate the whole.

